# Novel coronavirus disease 2019 (COVID-19) non-respiratory involvement

**DOI:** 10.1186/s43168-020-00030-1

**Published:** 2020-10-01

**Authors:** Eman Sobh, Einas Abuarrah, Khloud Gamal Abdelsalam, Sohaila Sabry Awad, Mohamed Ahmed Badawy, Mohamed A. Fathelbab, Mohamed Ahmed Aboulfotouh, Mohamed Fawzi Awadallah

**Affiliations:** 1 Chest Diseases Department, Faculty of Medicine for Girls, Al-Azhar University, Cairo, Egypt; 2 Respiratory Therapy Department, College of Medical Rehabilitation Sciences, Taibah University, Medina, Saudi Arabia; 3 Pharmaceutical Formulation and Development, Tubas, Palestine; 4 Biochemistry Unit, Chemistry Department, Faculty of Science, Damanhour University, Damanhour, Egypt; 5 Faculty of Science, Cairo University, Cairo, Egypt; 6 Cairo University Hospital, Cairo University, Cairo, Egypt; 7 Radio-diagnosis Department, Italy Military Hospital, Alexandria, Egypt; 8 Chest Diseases Department, Faculty of Medicine, Al-Azhar University, Damietta, Egypt

**Keywords:** COVID-19, Coronavirus, Extrapulmonary manifestations, SARS-CoV-2, Symptoms, Non-respiratory

## Abstract

**Background:**

Coronavirus disease 2019 (COVID-19) is a newly emerging pandemic that affected millions of people worldwide caused by novel coronavirus SARS-CoV-2. The first cases reported suffered from respiratory symptoms.

**Main body:**

Various extrapulmonary manifestations were linked to COVID-19 in several reports including cardiovascular, genitourinary, gastrointestinal, and skin. It is important that every clinician should be aware of these non-respiratory manifestations for early diagnosis and prompt management. This review aims to summarize the different extrapulmonary manifestations of COVID-19 disease and highlight the importance of multidisciplinary care.

**Conclusion:**

COVID-19 is a disease of multi-organ involvement. Manifestations may vary depending on which organ is involved.

## Background

Coronavirus disease 2019 (COVID-19) is caused by a novel single-strand ribonucleic acid (RNA) coronavirus known as severe acute respiratory syndrome coronavirus-2 (SARS-CoV-2). It has been recognized by the World Health Organization (WHO) as an international pandemic [1, 2]. SARS-CoV-2 primary attacks the lower respiratory system causing viral pneumonia, but it may also affect the heart, gastrointestinal system, liver, kidney, and central nervous system leading to multiple organ failure [3]. Several comorbidities have been identified as risk factors for severe COVID-19 disease. The most prevalent comorbidities detected in different studies were hypertension (HTN), diabetes mellitus (DM), respiratory diseases, and obesity [4–7]. In this review, we will through light on extrapulmonary manifestations caused by COVID-19 and their impact on outcome and case management which indicates a great need for multidisciplinary team.

The exact mechanism of extrapulmonary manifestations is still under research; several factors have been proposed either direct or indirect injury secondary to the inflammatory response to the viral injury [8, 9]. SARS-CoV-2 infection begins when the viral surface spike protein (S) binds to the human angiotensin-converting enzyme receptors (ACE2) receptor following activation of the spike protein by transmembrane protease serine 2 (TMPRSS2) that facilitates S protein priming [10]. ACE2 receptors are expressed in the lung (principally type II alveolar cells), heart, intestinal epithelium, kidney, vascular endothelium, and smooth muscle cells of all organs providing a mechanism for the COVID-19 multi-organ dysfunction [11–13].

Another mechanism is the severe inflammatory response induced by the viral infection in the lung as well as other organs [14]. Once inside the cells, SARS-COV-2 activates T lymphocytes triggering an intense immune response and an inflammatory reaction leading to an inflammatory cascade with the release of cytokines: interleukin (IL)-1, IL-6, tumor necrosis factor-α (TNF-α), granulocyte-macrophage colony-stimulating factor (GM-CSF), and interferon-γ (IFN-γ) known as cytokine storm resulting in tissue damage [15].

## Main text

### Cardiovascular (CV) manifestations

Recent studies found that cardiovascular comorbidities such as hypertension, coronary artery disease, and cerebrovascular disease are common in COVID-19 cases and are linked to severe disease, poor prognosis, and death [4–7, 16]. Besides, diabetes mellitus is a risk factor for heart failure as non-cardiovascular comorbidity [16]. Several existing reports suggested that SARS-CoV-2 infection leads to CV complications or exacerbation of preexisting cardiovascular disease (CVD) [5, 17]. Palpitations, cardiac arrhythmias, and cardiac arrest were common CV manifestations in patients with COVID-19 infection in one study [18]. Zhou and colleagues reported that cardiomyopathy and heart failure were observed in 23.0% of patients with COVID-19 disease [19]. Researchers found that among COVID-19-related deaths 11.8% of patients who did not have underlying cardiovascular disease had cardiac damage with elevated cardiac troponin I (catnip) or cardiac arrest during hospital admission [20]. Other researchers found elevated serum troponin levels in many patients infected with COVID-19, and it was associated with more severe disease and poor prognosis [21]. Another study showed that COVID-19 non-survivors had significantly higher D-dimer and fibrin degradation products (FDP) levels and longer prothrombin (PT) compared to survivors on admission [22].

Prevalent CVD may be a marker of accelerated immunologic dysregulation and aging that may relate indirectly to COVID-19 prognosis [21]. The exact mechanism of cardiac injury in COVID-19 is still under research. It was proposed that COVID-19 interacts with the cardiovascular system on multiple levels, increasing morbidity in patients with existing cardiovascular conditions and provoking myocardial dysfunction and injury [23]. The mechanism behind acute myocardial injury caused by SARS-CoV-2 infection might be related to human angiotensin-converting enzyme 2 receptor (ACE2) [20] which are highly expressed in the heart [11]. Besides, severe respiratory infection and hypoxia, especially in the setting of severe infection and acute respiratory distress syndrome (ARDS) due to COVID-19, it is likely that many patients will develop injuries such as myocardial injury, myocarditis, and acute coronary syndrome [17]. Meanwhile, pneumonia may cause significant ventilation perfusion mismatch, leading to hypoxemia, which significantly reduces the energy supply for cell metabolism, increases anaerobic fermentation, and leads to intracellular acidosis and oxygen free radicals that destroy the phospholipid layer of the cell membrane [24, 25]. Also, the high prevalence of arrhythmia might be attributable to hypoxia, metabolic disarray, and neurohormonal or inflammatory stress in the setting of viral infection in patients with or without prior CVD [26]. Abnormal coagulation resulting in venous thromboembolism and disseminated intravascular coagulopathy (DIC) are other contributing factors [22].

The results of previous reports indicate that cardiac injury, arrhythmia, and venous thromboembolism should be considered in any suspected or confirmed COVID-19 case and the patient should undergo a prompt clinical evaluation. Thromboprophylaxis should be added to therapy to those with severe disease.

### Gastrointestinal manifestations

There is evidence from previous studies that SARS-CoV-2 can invade the gastrointestinal (GI) tract through binding to ACE2 receptors where they are highly expressed as in the glandular cells of gastric, duodenal, ileal, colonic, and rectal epithelia; ileum; and colon [26–28]. ACE2 expression is rarely seen in esophageal mucosa, probably because the esophageal epithelium is mainly composed of squamous epithelial cells, which express less ACE2 than glandular epithelial cells [28]. Besides, genetic elements of the SARS-CoV-2 RNA had been found in feces [26–28]. Importantly, it is observed that 23.29% of patients continued to have positive results for SARS-CoV-2 in stool after showing negative results in respiratory samples [28]. This may raise the possibility of a fecal-oral transmission route and the GI can serve as a route of transmission [26–29].

Patients suspected to have COVID-19 presenting with GI symptoms such as nausea, diarrhea, and vomiting should be considered seriously [26]. SARS-CoV-2 may cause acute gastritis and enteritis, as evidenced by nausea, diarrhea, and vomiting [27]. Previous studies reported that most patients had at least one GI symptom, most commonly anorexia and diarrhea [26–30]. Patients with GI symptoms have a significantly high rate of fever, fatigue, headache, shortness of breath, and increased incidence of ARDS, and they also tend to have more severe/critical disease when compared with those without GI symptoms [25]. Electrolyte disturbances such as decreased sodium level secondary to GI manifestations contribute to the severity of the disease [25]. A study found that about 3% of patients admitted to the hospital presented with digestive symptoms without respiratory symptoms. They had a significantly longer time from onset of symptoms to hospital admission when compared with patients with no digestive symptoms [30]. Abnormal laboratory data in these patients included elevated levels of liver enzymes, alanine transaminase (ALT) and aspartate transaminase (AST) [25]. Significant prolongation of prothrombin time was found in patients with digestive symptoms, whereas other indicators of coagulation function were not significantly different [30]. Gastrointestinal manifestations may be attributed to the inflammatory response of SARS-CoV-2 in the gut, which may directly or indirectly damage the cells. Viral invasion of GI epithelial cells results in cytokine and chemokine release, activating acute intestinal inflammation characterized by infiltration of neutrophils, macrophages, and T cells. This inflammatory response is evidenced by diarrhea, elevated fecal calprotectin (FC), and systemic IL-6 response. SARS-CoV-2 infection exerts gut tropism characterized by an acute inflammatory response. This inflammatory process could deteriorate already present GI disorders like inflammatory bowel disease (IBD) [31]. In addition, the immunosuppressive drugs used to treat IBD may result in a more severe/critical course of COVID-19 [32].

Considering the previous points, GI symptoms should be evaluated promptly, and suspected COVID-19 infection should be considered to avoid complicated course and to prevent transmission to the community due to delayed diagnosis and treatment [33]. Meanwhile, both the SARS-COV-2 virus and the antiviral therapy can induce liver injury so continuous monitoring of liver functions is mandatory [15].

### Renal manifestations

Several studies reported acute kidney injury (AKI) in COVID-19 patients with variable incidence rate [24, 26, 32, 34, 35]. Worsening of preexisting chronic kidney disease (CKD) was also described in COVID-19 patients [36, 37]. Kidney injury is more linked to severe disease, old age, and comorbidities [26, 37], and it is a risk factor for poor prognosis [24, 38]. Markers of kidney injury observed in COVID-19 cases involved elevated serum creatinine and blood urea nitrogen, reduced glomerular filtration rate, proteinuria, and hematuria [35, 38, 39], in addition to decreased density of the kidney and edema as seen in computed tomography (CT) scanning of the involved patients [39].

The etiology of kidney involvement in COVID-19 patients is ambiguous, and several mechanisms are proposed: first, the COVID-19 virus utilizes the ACE2 receptor for entry to the cell as mentioned earlier [10, 11]. Human tissue RNA sequencing revealed that the ACE2 presence in the kidney is higher than that in the lung [39]. Therefore, the COVID-19 virus may attack renal epithelial cells [40]. This mechanism is verified by the presence of the virus RNA in the patients’ blood and urine [24]. Besides, a report of autopsies from patients showed severe acute tubular injury, inflammatory cell infiltration, arteriosclerosis, and accumulation of coronavirus-like particles in renal epithelial cells [37]. Second, kidney impairment may stimulate inflammation in the lung (collateral injury). Meanwhile, inflammation following lung injury may result in the deposition of immune complexes of viral antigen or virus-induced specific immunologic response leading to acute kidney injury [41, 42]. Third, the virus-induced cytokine storm may affect the kidney as well as other organs [14]. Hyaline thrombi with small vessel damage are found in the kidney of COVID-19 patients [43].

Prompt evaluation of kidney function is necessary in all cases of COVID-19 disease; continuous follow-up of renal function and proper management of any abnormal clinical finding should take place early to avoid deterioration.

### Reproductive manifestations

The effect of SARS-CoV-2 on the genital and reproductive system is still unknown. However, there are some reports describing testicular injury and possible impaired male fertility [44]. Viral markers have been found in the testis and semen raising the possibility of sexual transmission [45]. Significant changes in sex hormones in patients with COVID-19 were reported, which may be attributed to impaired gonadal function [46]. Mechanisms of the SARS-CoV-2 effect on the gonadal system are the same for other organs including ACE2 receptor binding and utilization of TMPRSS2 protein [8–10] which are highly expressed in the urogenital system [46]. The systemic and local inflammation process associated with the viral infection and the released cytokines, interferon, and inflammatory mediators contribute to the destructive effect [47, 48]. Therefore, continuous follow-up of the reproductive system is important in COVID-19 patients. There was no evidence for the presence of SARS-CoV-2 in amniotic fluid, cord blood, and breast milk samples. The intrauterine infection has not been reported [49].

### Neurologic manifestations

Neurologic symptoms have been reported in patients with COVID-19. The symptoms included central nervous system (CNS), peripheral nervous system (PNS), and skeletal muscle injury. The most common CNS symptoms were dizziness and headache, while the most common PNS symptoms were taste and smell impairment. Most neurologic manifestations occurred early in the illness (median time, 1–2 days) except for cerebrovascular disease and impaired consciousness. Some patients presented to the hospital with neurologic symptoms; even some had acute cerebrovascular attacks without any typical symptoms (fever, cough, anorexia, and diarrhea) of COVID-19 [50]. Several patients reported a partial or total loss of smell and/or taste [51, 52]. The extent of olfactory dysfunction is still not fully understood [52].

The neurologic manifestations can be due to SARS-COV-2 infection either directly or indirectly [13, 50]. SARS-COV-2 may enter the CNS through the hematogenous or retrograde neuronal route [53, 54]. The invasion of the nervous system occurs through binding to ACE2 receptors that are expressed in the nervous system and skeletal muscles [13, 50]. The researchers detected SARS-CoV-2 nucleic acid in the cerebrospinal fluid of those patients and in their brain tissue on autopsy [53, 54]. Specimens from patients with COVID-19 showed brain tissue hyperemia, edema, and degeneration of some neurons [55]. Immune suppression contributes to the neurologic injury indicated by the presence of a low number of lymphocytes in patients with CNS symptoms compared to those without CNS symptoms. Patients with severe infection had higher D-dimer levels than patients with the non-severe infection that makes patients with severe infection more likely to develop cerebrovascular disease [50]. The true magnitude of olfactory affection cannot be estimated because of the absence/scarcity of validated quantitative olfactory testing and neuroimaging performed for COVID-19 patients. The mechanism may be direct injury of the olfactory nerve or local inflammation of the nasal cavity [51, 52].

Therefore, we should pay close attention to neurologic manifestations in patients with COVID-19 disease especially those with severe disease, and any patient with unexplained neurologic deficits should be evaluated for SARS-CoV-2 infection.

### Cutaneous manifestations

A few cases with COVID-19 in China have been reported a skin rash, eczema, rosacea, atopic dermatitis, dryness, erythema, and urticaria [56, 57]. However, it is difficult to distinguish between the main causes of these symptoms [57]. Several factors can be behind skin manifestations including the immune response to infection, medications, or use of protective equipment in healthcare workers or even unknown [56–58]. Hundreds of healthcare workers in China were found to have skin symptoms on the hands, nose, cheeks, and forehead as a result of damage to the skin barrier due to the frequent use of disinfectants and prolonged wearing face masks, goggles, and gloves [57, 59–61]. Itching and skin infections can result from some drugs used to treat symptoms such as antimalarials, antivirals, and corticosteroids [57, 62]. Skin reactions may also appear as a form of the immune response against the fever [63, 64]. Although the relationship between COVID-19 and skin diseases is still mysterious and needs more clinical evidence, there is still some concern because the skin is the first line of defense for the immune system [57]. If one of these possibilities is correct, people with autoimmune disorders and chronic infections such as psoriasis, lupus, scleroderma, and vitiligo may be more susceptible to infection with the COVID-19 [61]. Therefore, the China Dermatological Society and the National Center for Clinical Research of Dermatology and Immunology have recommended taking preventive measures such as wearing goggles instead of a sanitary mask. Use foam-free cleaning products and skin moisturizing creams after cleaning your hands and wearing gloves that contain hyaluronic acid, ceramides, or vitamin E [65].

### Ocular manifestations

Ocular involvement in COVID-19 is uncommon, and conjunctival congestion was reported in less than 1% of cases [66]. Another study reported a higher rate of manifestations consistent with conjunctivitis (12 out of 38 patients, 31.6%) especially in cases with severe pneumonia. These manifestations involved conjunctival hyperemia, chemosis, epiphora, and increased secretions. Patients with eye manifestations had leukocytosis, neutrophilia, increased C-reactive protein (CRP), lactate dehydrogenase (LDH), and procalcitonin [67]. Conjunctival congestion may be an early manifestation in COVID-19 according to some case reports [67, 68].

Eye involvement may result from direct inoculation of the virus through respiratory droplets, migration through the nasolacrimal duct, or hematogenous spread [69]. The virus can also be transmitted through contaminated hands. The possibility of transmission of SARS-CoV-2 through ocular secretions is still unknown. Positive PCR for SARS-CoV-2 from conjunctival secretions was reported only in 2 cases in Wu study [67].

Ophthalmologists should be aware of the possibility of eye involvement in COVID-19 and should take infection control precautions and refer suspected cases for specialists to be evaluated thoroughly.

### Endocrinal manifestations

The relation between blood sugar and pulmonary diseases is well established [70]. Studies found that COVID-19 may cause elevation of blood sugar in diabetics and some experience poor control [71]. A preliminary data from Sebeokian et al. study found that more than half of all cases confirmed or suspected COVID-19 had hyperglycemia and nearly one third had diabetic ketoacidosis. In addition, some cases developed new-onset diabetes [72]. Direct pancreatic involvement by SARS-CoV-2 was suspected in severe COVID-19 cases due to the high expression of ACE2 receptors in the pancreas, in addition to severe inflammatory reaction which may explain pancreatic failure [73]. This may be associated with high serum amylase and/or lipase levels [74]. So, strict control and follow-up of blood sugar in all cases of suspected or confirmed COVID-19 is important to avoid flaring of infection and/or complications of uncontrolled diabetes. In severe cases, a panel of endocrine markers should be evaluated.

## Conclusion

Every organ in the body is susceptible to injury caused by the SARS-CoV-2 virus, and at the current time, several symptoms may be attributed to COVID-19 disease. A high index of suspicion and prompt evaluation of every case is mandatory to avoid deterioration and complications. Follow-up of any organ affected should be carried out after recovery, and early management of complications is warranted. At the same time, clinicians not involved in the management of COVID-19 should be aware of the possibility of other routes of possible transmission and take infection control precautions until the situation is clear. Multidisciplinary care is important to avoid complications and proper control of any organ affection.

## Data Availability

Not applicable

## Abbreviations

RNA: Ribonucleic acid
SARS-CoV-2: Severe acute respiratory syndrome coronavirus-2
WHO: World Health Organization
COVID-19: Coronavirus disease 2019
HTN: Hypertension
DM: Diabetes mellitus
ACE: Angiotensin-converting enzyme
IL: Interleukin
TNF- α: Tumor necrosis factor-α
GM-CSF: Granulocyte-macrophage colony-stimulating factor
IFN-γ: Interferon-γ
FDP: Fibrin degradation products
PT: Prothrombin time
CVD: Cardiovascular disease
ARDS: Acute respiratory distress syndrome
GI: Gastrointestinal
ALT: Alanine transaminase
AST: Aspartate transaminase
FC: Fecal calprotectin
IBD: Inflammatory bowel disease
CKD: Chronic kidney disease
CNS: Central nervous system
PNS: Peripheral nervous system
CRP: C-reactive protein
LDH: Lactate dehydrogenase
